# Strength Recovery of Thermally Damaged High-Performance Concrete during Recuring

**DOI:** 10.3390/ma17143531

**Published:** 2024-07-17

**Authors:** Ye Li, Haodong Wang, Hangqi Lou

**Affiliations:** 1 The National Key Laboratory of Water Disaster Prevention, Nanjing Hydraulic Research Institute, Nanjing 210098, China; liye@hit.edu.cn; 2 School of Civil and Environmental Engineering, Harbin Institute of Technology, Shenzhen 518055, China; wanghdhitsz@163.com

**Keywords:** high-performance concrete, post-fire curing, carbonation

## Abstract

High-performance concrete (HPC) experiences significant degradation in its mechanical properties after fire exposure. While various post-fire curing methods have been proposed to rehabilitate thermally damaged concrete (TDC), the physical and chemical changes occurring during these processes are not well-understood. This study examines the strength and microstructure restoration of HPC through water and water–CO_2_ cyclic recuring. HPC samples were initially heated to 600 °C and 900 °C, then subjected to water and cyclic recuring. Results indicate that the mechanical performance recovery of thermally damaged HPC is significantly better with cyclic recuring than with water recuring. The compressive strength of HPC samples exposed to 600 °C and 900 °C reached 131.6% and 70.3% of their original strength, respectively, after cyclic recuring. The optimal recuring duration for substantial recovery in thermally damaged HPC was determined to be 18 days. The strength recovery is primarily due to the healing of microcracks and the densification of decomposed cement paste. These findings clarify the physical and chemical processes involved in post-fire curing of HPC, highlighting the potential of water and water–CO_2_ cyclic recuring in the rehabilitation of TDC.

## 1. Introduction

Concrete is widely recognized for its inherent fire-resistant properties; however, its mechanical performance significantly declines when exposed to high temperatures. Elevated temperatures lead to the decomposition of hydration products, coarsening of the pore structure, thermal cracking, and mineralogical phase transformations, all of which contribute to the degradation of concrete’s mechanical properties [1,2,3]. The compressive strength of concrete deteriorates rapidly under high-temperature conditions, leaving post-fire structures with limited options: demolition and rebuilding or repair and reinforcement. Reinforcing post-fire structures is typically both time-consuming and labor-intensive [4,5]. Crook et al. [6] demonstrated in 1970 that concrete subjected to a temperature of 620 °C could regain its compressive strength through water recuring. Since then, researchers have concentrated on exploring the self-healing capability of concrete after fire damage, aiming to develop more efficient repair techniques to enhance the safety and reliability of post-fire concrete structures [7,8,9,10,11,12,13,14].

Concrete decomposes through various mechanisms when exposed to high temperatures. These processes include the breakdown of monosulfoaluminate (AFm) and ettringite (AFt) between 110 and 150 °C, the decomposition of calcium hydroxide (Ca(OH)_2_, CH) from 400 to 500 °C, and the dehydration and subsequent decomposition of the C-S-H gel occurring between 200 °C and 900 °C [1,15,16,17]. Through techniques such as X-ray diffraction analysis and scanning electron microscopy, Alonso et al. [18] and Wang et al. [19] found that the decomposition products of the C-S-H gel, CH and AFt can rehydrate to form new compounds during the recuring of TDC [20,21]. These rehydration products play a crucial role in repairing microcracks and coarsened pores caused by high temperatures, thereby contributing to the restoration of concrete’s compressive strength [22,23,24,25]. Yim et al. [26] observed a significant improvement in the mechanical properties of TDC when cured in high-humidity environments or immersed in water, attributable to the increased formation of rehydration products. Therefore, the selection of a recuring method [26], the reactivity of rehydration phases [27], and the extent of high-temperature degradation [13] are all critical factors that influence the effectiveness of recuring TDC.

Poon et al. [28] conducted experiments on HPC containing silica fume (SF) and fly ash (FA), exposed it to high temperatures, and subsequently subjected it to water recuring. Their findings highlighted that blended concrete exhibited superior recovery during recuring compared to ordinary Portland cement concrete. The high-reactivity SiO_2_ in SF and FA acted as a pozzolan, reacting with CH in HPC during recuring, and leading to increased formation of C-S-H gel, which effectively filled microcracks and coarsened pores caused by thermal damage, thereby enhancing the mechanical properties of the concrete [15,29]. Moreover, the consumption of CH in this process helped prevent potential secondary damage from excessive CH generation in HPC with a high CaO content [30]. However, incorporating high-reactivity SiO_2_ in HPC also resulted in the formation of phases with lower calcium-to-silica ratios, such as γ-C_2_S, CS, and C_3_S_2_, under high-temperature conditions, which reduced the recovery potential of HPC during water curing [31]. These phases, prevalent in thermally damaged HPC, exhibit higher carbonation activity than hydration activity [27,32,33]. Addressing this, Li et al. [22] proposed a water–CO_2_ cyclic recuring method for thermally damaged HPC, demonstrating superior recovery performance compared to water recuring after exposure to temperatures of 800 °C and above.

Existing research has predominantly focused on the long-term recovery of mechanical properties in thermally damaged HPC during curing, with limited attention to the efficiency of strength recovery across different curing durations and the underlying microstructural mechanisms. Addressing these gaps, this study aims to elucidate the strength development process and its micro-mechanisms during water and water–CO_2_ recuring of HPC. HPC samples, incorporating SF as a supplementary cementitious material, were exposed to temperatures of 600 and 900 °C, followed by water or cyclic recuring for up to 30 days. Various characterization methods were employed at 3-, 6-, 18-, and 30-day intervals to explore the relationships between compressive strength, changes in phase composition, and microstructural development during the recuring process. This study investigates influencing factors and mechanisms contributing to strength recovery in thermally damaged HPC, identifying optimal recuring regimes and durations for effective strength recovery. Section 2 of this paper will detail the experimental materials, sample preparation, procedures, and characterization methods employed. Section 3 will analyze results from compressive strength tests, X-ray diffraction, scanning electron microscopy, and mercury intrusion porosimetry. Section 4 will discuss experimental findings, compare them with previous research, and summarize conclusions.

## 2. Materials and Methods

### 2.1. Materials and Sample Preparation

The mixture proportions for the HPC samples are detailed in Table 1. The cementitious components consist of CEM I 52.5 Portland cement produced by Huarun Cement Co., Ltd. located in Shenzhen, China, and SF97 silica fume produced by Linyuan Microsilica Co., Ltd. located in Xi’an, China, with silica fume substituting 10% of the cement by mass. The chemical compositions of the cement and silica fume are listed in Table 2. For the preparation of the HPC samples, silica flour and sieved standard quartz sand were employed as aggregates. The particle size of the silica flour and the sieved standard quartz sand ranged from 15 to 293 μm and 0.08 to 1.18 mm, respectively. The particle size distribution (D10, D50, D90) of the materials is presented in Table 3. The water-to-binder ratio for the HPC samples was set at 0.36. To achieve the desired consistency and workability, 6.1 kg/m^3^ of a polycarboxylate superplasticizer, produced by Master Builders Solutions located in Shanghai, China, with a 24% solid content was added. The technical properties of the superplasticizer are presented in Table 4. Additionally, to prevent explosive spalling during high-temperature exposure, 3.0 kg/m^3^ of polypropylene (PP) fibers with a length of 12 mm and a diameter of 31 μm were incorporated into the HPC samples. The samples, referred to as “mortar”, are labeled as “M” in subsequent figures in the Results and Discussion Sections.

The HPC material was prepared using a 20 L mortar mixer. To prevent powder overflow during mixing, the dry materials were added in the following sequence: cement, silica fume, silica flour, and standard quartz sand. These dry components were mixed at 140 rpm for 3 min to ensure uniformity. Subsequently, a mixture of superplasticizer and water was added, and the blend was mixed at 140 rpm for 3 min. Once a homogeneous mix was achieved, PP fibers were introduced, and the mixture was stirred at 285 rpm for 6 min.

After mixing, the HPC material was poured into 50 × 50 × 50 mm^3^ cubic molds in two layers. The first layer was evenly spread using a spreader positioned vertically at the top of the mold. The mold was then placed on a vibrating table and vibrated 60 times to ensure proper compaction. Next, the second layer of mortar was added, spread evenly, and subjected to 60 vibrations as well. After vibrating, the mold was removed, and the excess material extending beyond the mold edges was scraped off using a metal ruler. Finally, the surface of the samples was smoothed to ensure uniformity. The compaction procedure of the HPC samples follows the ISO 679-2009 standard [34]. Following initial setting, the samples were covered with plastic film to prevent surface shrinkage cracking and left to stand for 24 h. After this period, the samples were demolded and submerged in saturated limewater at 20 ± 3 °C for 89 days. Following the curing period, the samples were removed for subsequent testing.

### 2.2. Heating and Recuring Regimes

**Figure 1:**
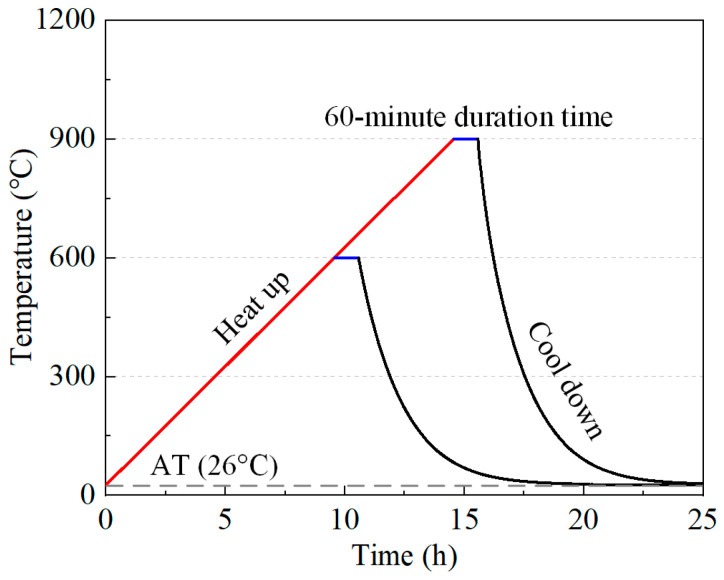
Heating and cooling curve of the electrical furnace.

After a 90-day curing period, the samples were heated to temperatures of 600 and 900 °C at a rate of 1 °C/min in a FO811C muffle furnace produced by Yamato Scientific Co., Ltd. located in Tokyo, Japan. Upon reaching the desired temperature, the samples were maintained at this temperature for 1 h to ensure uniform temperature distribution. Subsequently, the samples were allowed to cool naturally to ambient temperature inside the furnace. The heating and cooling curve is depicted in Figure 1.

**Figure 2:**
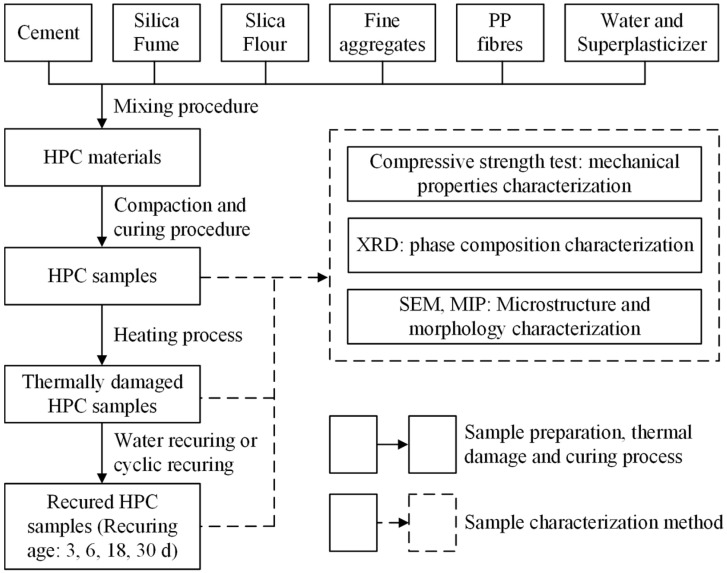
Research content and process.

For water (W) recuring, the heated samples were immersed in lime-saturated water for 3, 6, 18, and 30 days. For water–CO_2_ cyclic (C) recuring, the heated samples were submerged in lime–saturated water for 3 days, then transferred to an environmental chamber for another 3 days. The chamber conditions were set at 30 ± 1 °C, 40 ± 1% relative humidity, and 20 ± 0.2% CO_2_ concentration. Low relative humidity was selected to favor CO_2_ diffusivity and enhance carbonation efficiency in an unsaturated micropore solution [35,36]. The cyclic recuring regime was applied for 3, 6, 18, and 30 days. The experimental and research processes, as well as the sample testing methods used in this study, are illustrated in Figure 2.

### 2.3. Characterization Methods

#### 2.3.1. Compressive Strength

Compressive strength tests on the HPC samples were performed in accordance with ASTM C109/C109M-11 [37]. Following high-temperature exposure and the specified recuring periods, the samples were tested using a 600 kN universal testing machine. A constant loading rate of 2.4 kN/s was applied until the samples failed, and the maximum load was recorded. Three HPC samples were tested for each condition. The standard deviation of the strength values of the three cubic samples was calculated and represented as error bars in the strength graphs.

#### 2.3.2. X-ray Diffraction

X-ray diffraction (XRD) analysis was carried out using a Bruker D8 Advance X-ray powder diffractometer produced by Bruker Corporation located in Billerica, MA, USA. XRD patterns were obtained for the HPC samples before heating, after heating, and during the recuring. Fitting analysis was conducted to identify phases and their relative proportions. Post-recuring HPC samples were collected from the surface to a depth of 10 mm, then ultrasonically cleaned and soaked in isopropanol for 24 h to halt further hydration. Samples were dried in a vacuum oven at 40 °C, ground into a fine powder, and passed through a 200-mesh sieve. The internal standard method was employed for phase proportion analysis, with ZnO incorporated into the powdered samples at a 10% mass ratio. The mixture was homogenized using a mortar and pestle for 15 min. XRD scanning was performed from 5° to 65° at a rate of 2°/min. Qualitative and quantitative analyses of the XRD patterns were conducted using HighScore Plus.

#### 2.3.3. Scanning Electron Microscopy

Scanning electron microscopy (SEM) tests were performed using a Phenom ProX G6 SEM produced by Thermo Fisher Scientific Inc. located in Waltham, MA, USA to observe microstructural changes in the HPC samples. Samples for SEM tests were taken from a depth of 3 to 8 mm from the surface. After extraction, the samples were pre-polished with 600-grit sandpaper, then soaked in isopropanol for 24 h to halt hydration. The samples were dried in a vacuum oven at 40 °C, embedded in epoxy resin, and allowed to harden for 24 h. The hardened samples were polished with successive grades of sandpaper, 600-grit, 1200-grit, 2000-grit, and 1 μm diamond paste on a Buehler TexMet P polishing cloth using a MoPao4S automatic grinding and polishing machine produced by Weiyi Experiment Machine Manufacturing Co.,Ltd. located in Laizhou, China. To enhance conductivity, a gold sputter coating was applied for 40 s using a Zhongke SBC-12 ion sputtering instrument. Images were captured using a BSE imaging detector at ×1000 magnification and 15 kV acceleration voltage.

#### 2.3.4. Mercury Intrusion Porosimetry

Mercury intrusion porosimetry (MIP) was conducted using a Micromeritics Autopore IV 9500 porosimeter produced by Micromeritics Instrument Corporation located in Norcross, GA, USA to measure pore structure distribution in the HPC samples. Samples for MIP testing were taken from a depth of 3 to 8 mm, ultrasonically cleaned, soaked in isopropanol for 24 h, and dried in a vacuum oven at 40 °C. MIP tests covered a pore diameter range from 5 nm to 360 μm, with a maximum pressure of 33,000 psi.

## 3. Results and Discussion

### 3.1. Compressive Strength

**Figure 3:**
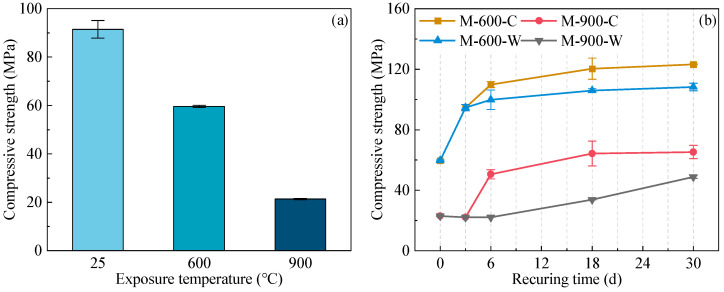
Compressive strength of the HPC samples (**a**) after heating at various temperatures; (**b**) at various recuring ages after exposure to 600 °C and 900 °C.

Figure 3a illustrates the compressive strength of the HPC samples after exposure to elevated temperatures. The results indicate the compressive strength of the HPC samples decreased to 65.2% and 22.3% of their original values after exposure to 600 and 900 °C, respectively. The decline in strength at 600 °C can be attributed to the decomposition of hydration products, such as AFt and CH, alongside dehydration and recrystallization of the C-S-H gel. These processes lead to pore structure coarsening and microcrack formation [38,39]. At 900 °C, extensive decomposition of the C-S-H gel causes significant shrinkage of the cement paste and further coarsening of the pore structure, resulting in a severe loss of compressive strength.

Figure 3b depicts the recovery of compressive strength of thermally damaged HPC during the recuring process. For HPC samples damaged at 600 °C, their compressive strength can be restored to pre-damage levels through recuring. After water recuring, the compressive strength reached 108.3 MPa, while cyclic recuring resulted in a compressive strength of 123.3 MPa, which is 13.9% higher compared to water recuring. This enhanced recovery during cyclic recuring is attributed to the carbonation process, which further improves the micro-mechanical properties of the HPC samples. In the initial stages of recuring for samples damaged at 600 °C, their compressive strength developed rapidly, although the rate of strength recovery decreased over time. By the 18th day of both water and cyclic recuring, compressive strength had recovered to 95.0% of the total recovery observed at 30 days.

For HPC samples damaged at 900 °C, there was minimal recovery in compressive strength during the initial 6 days of water recuring; however, the mechanical properties of the HPC gradually improved from the 6th to the 30th day, ultimately achieving a compressive strength of 48.9 MPa. During cyclic recuring, there was a notable increase in compressive strength during the initial carbonation phase from the 3rd to the 6th day. The first cycle of cyclic recuring showed the most substantial increase, reaching 97.7% of the total recovery after three recuring cycles. After 30 days of cyclic recuring, the compressive strength of the HPC samples reached 65.3 MPa, marking a 33.5% increase compared to the samples subjected to water recuring. Detailed analysis of the changes in phase assemblage and the microstructure of the HPC samples across different recuring durations is provided in the subsequent sections.

### 3.2. Phase Assemblage

The XRD test results were qualitatively analyzed using HighScore Plus software v3.0.5. CIF files of the phases present in the samples were imported into the software for quantitative analysis. Given that 10% ZnO powder was intentionally added to the samples, the internal standard method was employed to determine the content of crystalline and amorphous phases in the samples. The XRD patterns and quantitative XRD (QXRD) results were plotted using Origin, and the phases in the XRD patterns were labeled using Visio.

**Figure 4:**
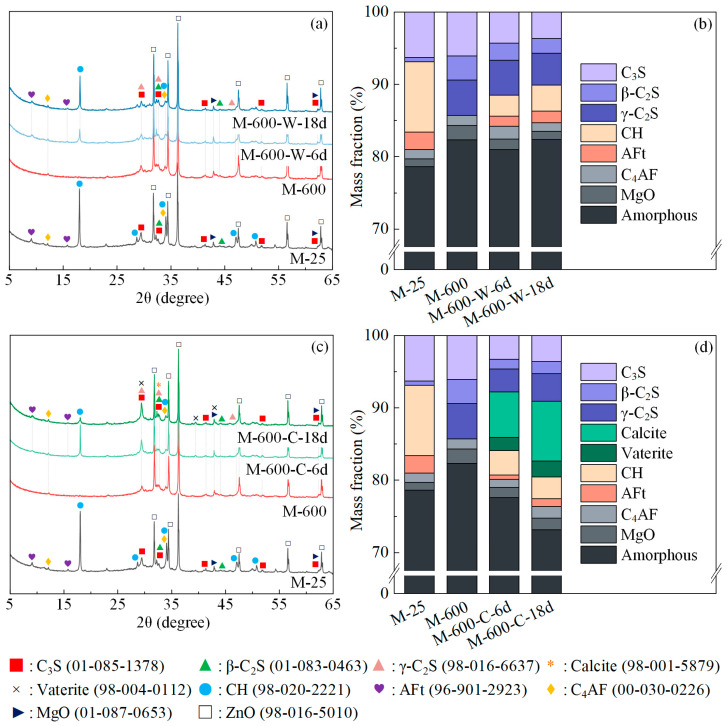
The XRD patterns and QXRD results of the samples exposed to 600 °C at various recuring stages: (**a**,**b**) water recuring; (**c**,**d**) cyclic recuring.

Figure 4 illustrates the XRD patterns and QXRD results of the HPC samples after exposure to 600 °C and during the recuring process. Following exposure to 600 °C, the diffraction peaks of AFt and CH disappeared completely, while the C_3_S peaks between 28° and 33° decreased, and a γ-C peak became evident. QXRD analysis revealed the presence of 4.9% γ-C_2_S in the sample M-600, attributed to the partial decomposition of the C-S-H gel. This decomposition led to the formation of β-C_2_S, which further transformed into γ-C_2_S below 500 °C [40].

After 6 days of water recuring (Figure 4b), the content of C_3_S and β-C_2_S in the HPC samples decreased by 1.8% and 0.9%, respectively. Dehydration products and unhydrated cement rehydrated to form 2.9% CH and 1.4% AFt. Between 6 and 18 days of the water recuring, the rate of rehydration product formation declined, with only an additional 0.6% CH and 0.4% AFt formed during this period. This trend aligns with the compressive strength development, which also shows a gradual decrease in the rate of increase as the recuring period extends.

For the sample M-600-C-6d, the carbonation process led to the generation of 6.3% calcite and 1.8% vaterite after one cycle of cyclic recuring. From day 6 to 18 of the cyclic recuring, an additional 2.0% calcite and 0.4% vaterite were generated, indicating a significant decrease in the rate of carbonation product formation. Comparing the XRD patterns of M-600-C-6d and M-600-C-18d, a noticeable decrease in the peak intensity of the rehydration product CH is observed as the duration of cyclic recuring increases. This suggests that the rehydration product CH is consumed by carbonation during cyclic recuring. Furthermore, comparing the XRD patterns of M-600-C-18d and M-600-W-18d, the amorphous phase content decreased after cyclic recuring, indicating that carbonation transformed the C-S-H gel into CaCO_3_ and silica gel [41,42,43]. During cyclic recuring, carbonation promoted further rehydration of unhydrated cement by consuming hydration products, leading to an increase in γ-C_2_S consumption and accelerating the consumption rate of β-C_2_S.

**Figure 5:**
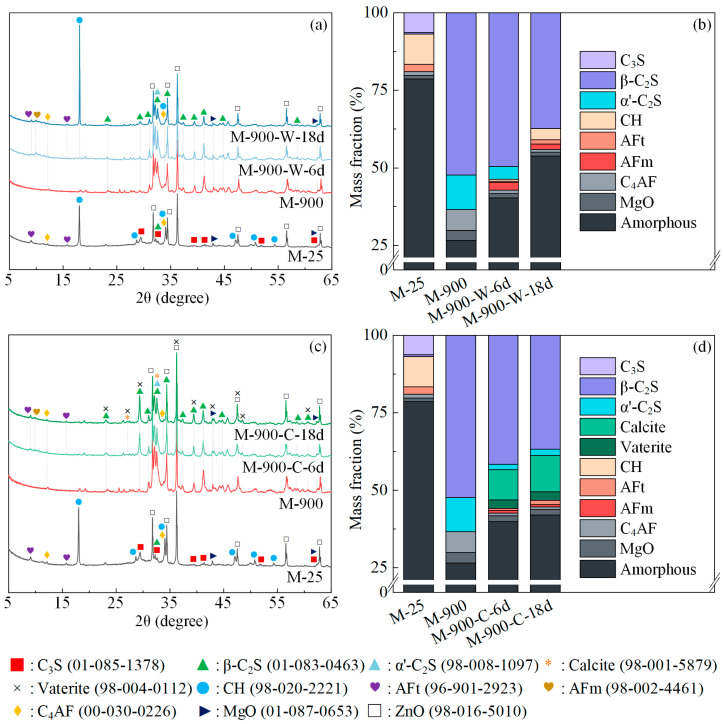
The XRD patterns and QXRD results of the samples exposed to 900 °C at various recuring stages: (**a**,**b**) water recuring; (**c**,**d**) cyclic recuring.

Figure 5 shows the XRD patterns and QXRD results of the HPC samples after exposure to 900 °C and during the recuring process. After exposure to 900 °C, the amorphous phase content in the HPC samples significantly decreased. The C-S-H gel underwent extensive decomposition, resulting in the formation of a considerable amount of β-C_2_S, while β-C_2_S partially transformed into α’-C_2_S above 690 °C [44,45]. The C_3_S completely disappeared post 900 °C exposure due to the solid-phase reaction between C_3_S and high-silica content phases, leading to the formation of low-calcium silicate β-C_2_S at high temperatures [46,47]. After exposure to 900 °C, the β-C_2_S content in the HPC samples increased by 51.1%, and 10.9% α’-C_2_S was generated.

During water recuring of the HPC samples damaged at 900 °C, α′-C_2_S rapidly rehydrated to generate C-S-H gel and CH. Concurrently, CH reacted with silica fume to form more C-S-H gel, increasing the amorphous phase content in the HPC samples. After 6 days of water recuring (Figure 5b), 0.7% CH, 0.4% AFt, and 2.5% AFm were formed, and the amorphous phase content increased by 14.2%. Between 6 and 18 days, β-C_2_S slowly hydrated, further increasing the amorphous phase and CH content in HPC. During this period, the CH content in HPC rose by 2.9%. However, the main generation of AFt and AFm occurred in the first 6 days of water recuring.

Following the first cycle of cyclic recuring for HPC samples damaged at 900 °C, 9.8% calcite and 2.7% vaterite were generated, alongside the rehydration products AFm and AFt. From day 6 to 18 of the cyclic recuring, the rate of CaCO_3_ formation significantly decreased, with only an additional 2% calcite formed during this period. Comparing the XRD patterns of M-900-C-6d and M-900-W-6d, an increase in β-C_2_S consumption and a lower amorphous phase content were observed in the HPC samples after cyclic recuring, indicating the promoting effect of carbonation on β-C_2_S and C-S-H.

### 3.3. Microstructure Observations

**Figure 6:**
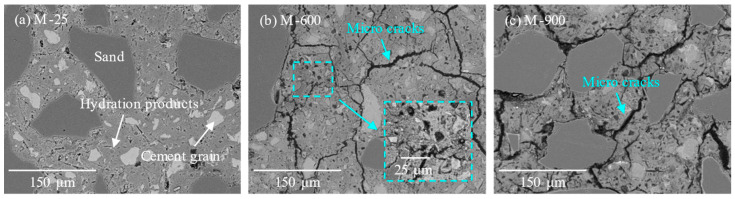
The microstructure of the HPC samples: (**a**) before heating; (**b**) after exposure to 600 °C; (**c**) after exposure to 900 °C.

The microstructure of HPC samples after heat exposure and subsequent recuring was examined using backscattered electron (BSE) imaging mode on an SEM. Some of the BSE images have been magnified by a magnification of 2000× and are displayed within blue–green dashed boxes to clearly illustrate the changes in the pore structure. The micrographs of the rehydration and carbonation products during the recuring process are presented within an orange dashed box. Visio was used to compile and integrate the SEM images. Figure 6 provides SEM-BSE images depicting the microstructure of HPC at ambient temperature and following exposure to temperatures of 600 and 900 °C. In the BSE images, the larger and more uniform entities are identified as sand grains. The cement paste region consists of brighter areas representing unhydrated cement particles and gray areas representing hydration products. Darker regions indicate the presence of pores or microcracks. Before exposure to high temperatures, HPC samples exhibited a dense microstructure with minimal pores present in the matrix, primarily due to hydration shrinkage.

After exposure to 600 °C, the HPC samples experienced dehydration shrinkage of the cement paste, thermal expansion of aggregates [48], and numerous microcracks induced by thermal stress. Additionally, the decomposition of hydration products such as AFt and CH, along with the dehydration and recrystallization of the C-S-H gel [39], exacerbated the deterioration of the HPC matrix. The formation of microcracks significantly reduced the compressive strength of the HPC samples. After exposure to 900 °C, significant decomposition of the C-S-H gel and phase transformations of unhydrated cement particles, along with surrounding phases, further intensified the microstructural damage of HPC. This led to additional coarsening of the pore structure and a significant increase in the number and size of microcracks within the matrix (Figure 6c).

**Figure 7:**
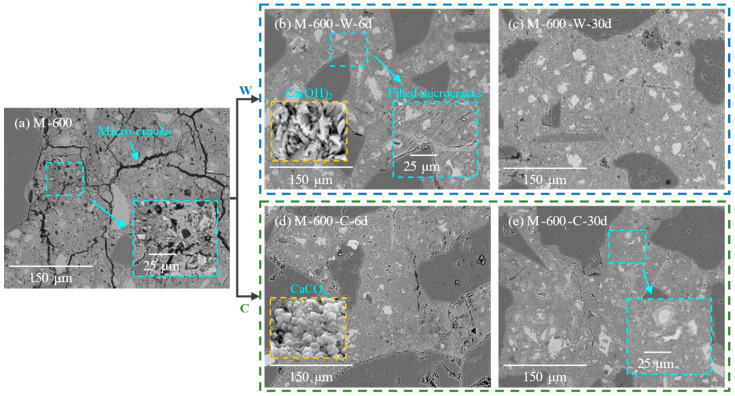
Microstructure of HPC samples thermally damaged at 600 °C: (**a**) before recuring; (**b**,**c**) at various recuring ages during water recuring; (**d**,**e**) at various recuring ages during cyclic recuring.

Figure 7 illustrates the microstructural changes in the HPC samples damaged at 600 °C during the recuring process at different stages. The dense pore network within HPC, formed due to high-temperature exposure, facilitated water penetration into the sample. During water recuring, dehydration products and unhydrated cement rehydrated to form CH, AFt, and new C-S-H gel, which filled the pores induced by high-temperature degradation. After 6 days of water recuring, a substantial amount of CH crystals was observed filling the microcracks (Figure 7b). This filling of rehydration products, along with matrix swelling due to water absorption, significantly increased the compactness of the matrix. From day 6 to 30 of the water recuring, there was no significant change in the compactness of the HPC matrix (Figure 7c), which was consistent with the compressive strength development.

During the cyclic recuring of the HPC samples damaged at 600 °C, rehydration products such as CH and C-S-H underwent carbonation, leading to the formation of calcium carbonate and silica gels within the matrix, which possess better mechanical properties (Figure 7d) [49,50,51,52]. Calcium carbonate interacted with surrounding substances in the HPC matrix through ionic and covalent bonds [53]. Additionally, the formation of high-polymerization silica gel and calcium carbonate crystals effectively reduced the porosity of the microstructure, significantly improving the micro-mechanical properties (indentation modulus and hardness) of the carbonated cement paste [54,55]. This enhancement contributed to the overall mechanical properties of the HPC samples.

**Figure 8:**
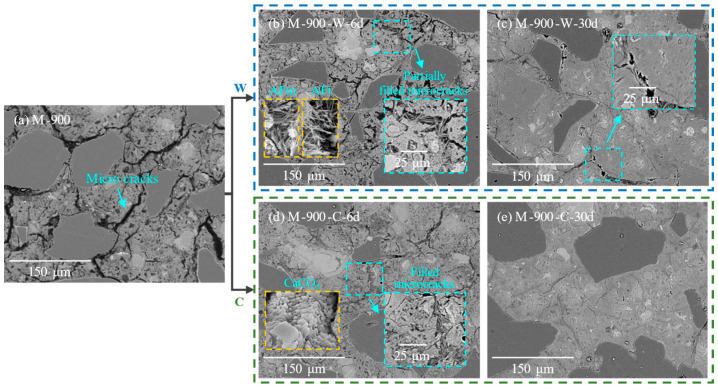
Microstructure of HPC samples thermally damaged at 900 °C: (**a**) before recuring; (**b**,**c**) at various recuring ages during water recuring; (**d**,**e**) at various recuring ages during cyclic recuring.

Figure 8 shows the microstructural changes in HPC samples after exposure to 900 °C and during the recuring process. In the initial 6 days of water recuring, a significant amount of AFm and AFt crystals were observed filling the microcracks (Figure 8b); however, due to the presence of numerous pores between the AFm and AFt crystals and the limited improvement in the matrix compactness, the compressive strength of the HPC samples did not increase significantly. From day 6 to 30 of the water recuring, β-C_2_S continued to hydrate, generating additional CH and C-S-H gel that filled the coarsened pores and a few microcracks, leading to a marked improvement in the compactness of the HPC matrix (Figure 8c) and, consequently, an increase in its compressive strength.

After 6 days of cyclic recuring for HPC samples damaged at 900 °C (Figure 8d), calcium carbonate crystals were observed filling the pores between the AFm and AFt crystals within the microcracks of the HPC samples. This increased the compactness of the HPC matrix. The filling of pores by calcium carbonate contributed to the enhancement of the compressive strength of the HPC samples. As cyclic recuring continued from day 6 to 30, further hydration and carbonation occurred, leading to the formation of additional rehydration and carbonation products that filled the pores (Figure 8e). This process increased the compactness of the HPC matrix and consequently improved its mechanical properties.

### 3.4. Porosity and Pore Size Distribution

The porosity of the HPC samples is categorized into three ranges: microcracks or coarse pores (>1 μm); large capillaries (50 nm to 1 μm); and small capillaries (6 to 50 nm) [56,57,58]. The results of the MIP tests and the corresponding pore classification were plotted using Origin.

**Figure 9:**
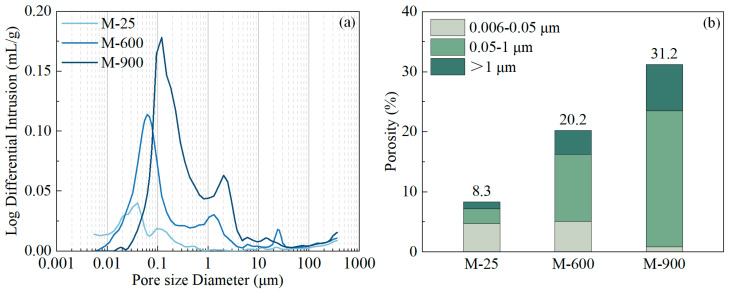
The pore size distributions and porosity categorized into three ranges for HPC samples after heating at various temperatures: (**a**) pore size distributions; (**b**) porosity.

Figure 9 depicts the changes in porosity of the HPC samples at ambient temperature and after exposure to high temperatures. The HPC samples exhibited a high degree of compactness with a unimodal pore distribution peaking at a pore diameter of 40 nm. After exposure to 600 °C, the pre-existing pores in the HPC samples coarsened, resulting in a trimodal pore distribution. Both the diameter and peak value of the initial pore distribution increased, and new peaks emerged at 1.3 and 24.0 μm. These peaks correspond to the microcracks generated in the matrix and pores formed from the melting of PP fibers, respectively (Figure 9a). The increase in porosity, especially in coarse capillary pores and microcracks due to thermal damage, resulted in a doubling of the total porosity compared to the initial state. This rise in porosity is a key factor contributing to the reduction in compressive strength [22]. Following exposure to 900 °C, the pore structure further coarsened, with the pore distribution still exhibiting three peaks but shifting to coarser sizes of 0.1, 5.0, and 21.0 μm (Figure 9a). The total porosity of the HPC samples increased by a factor of 3.8 compared to ambient temperature conditions, leading to a substantial decrease in compressive strength.

**Figure 10:**
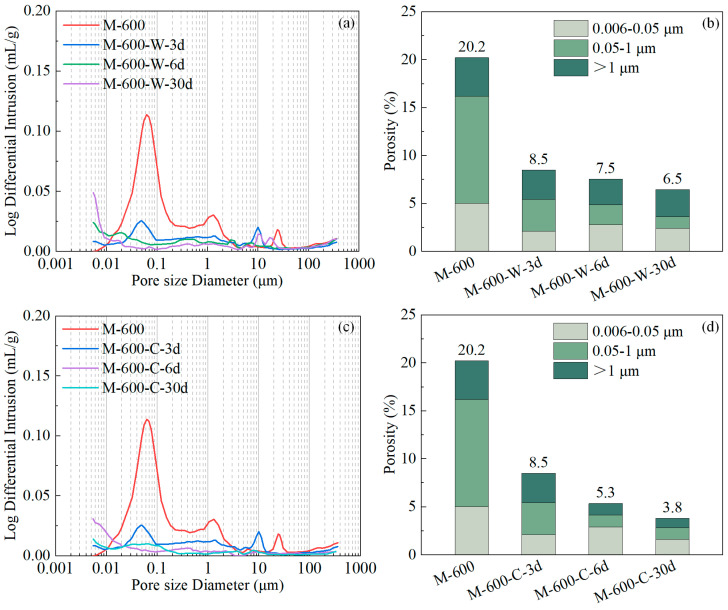
The pore size distributions and porosity categorized into three ranges of HPC samples exposed to a temperature of 600 °C at various recuring stages: (**a**,**b**) water recuring; (**c**,**d**) cyclic recuring.

Figure 10a and c present the changes in pore size distribution for the HPC samples damaged at 600 °C during the water and cyclic recuring processes, respectively. The corresponding pore volume distribution charts are shown in Figure 10b,d. During the water recuring of the HPC samples damaged at 600 °C, the rehydration rate of dehydration products was high, and the matrix swelled due to water absorption, which significantly reduced the porosity of the HPC. After 3 days of water recuring, the peak pore size of the original distribution decreased, and the peak value was lowered (Figure 10a). The porosity of microcracks, large capillaries, and small capillaries was reduced by 1.0%, 7.8%, and 2.9%, respectively. This increase in matrix compactness enhanced the mechanical properties of the HPC samples. From day 3 to 6 of the water recuring, the rate of porosity reduction slowed, with no significant peaks in the pore size distribution chart, indicating a refinement of the pore structure and further improvement in the mechanical properties of the HPC samples. From day 6 to 30 of the water recuring, the rate of porosity reduction significantly decreased, with the total porosity of the HPC decreasing by only 1.0% during this period; consequently, the matrix compactness did not further increase, leading to almost no improvement in the compressive strength of the HPC samples.

During cyclic recuring of the HPC samples damaged at 600 °C, the carbonation process further densified the HPC matrix. Dehydration products rehydrated to fill microcracks and coarsened pores, while rehydration products and existing C-S-H gel underwent carbonation, forming calcium carbonate, silica gel, and other substances. This process further reduced the porosity between the gels [55]. After one cycle of cyclic recuring, the total porosity of the HPC samples (M-600-C-6d) was lower than that of the HPC samples after 6 days of water recuring (M-600-W-6d) (Figure 10b,d). This increase in matrix compactness significantly enhanced the mechanical properties of the HPC samples, with the improvement being more pronounced than in HPC samples subjected to water recuring.

**Figure 11:**
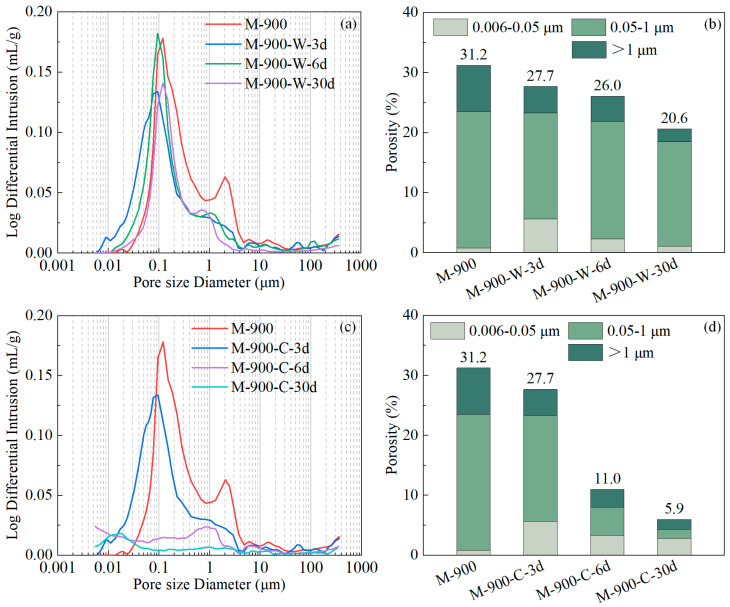
The pore size distributions and porosity categorized into three ranges of HPC samples exposed to a temperature of 900 °C at various recuring stages: (**a**,**b**) water recuring; (**c**,**d**) cyclic recuring.

Figure 11a,c present the changes in pore size distribution for the HPC samples damaged at 900 °C during water and cyclic recuring processes, respectively. The corresponding pore volume distribution charts are shown in Figure 11b,d. During the first 6 days of water recuring (M-900-W-6d), the formation of AFm and AFt crystals filled the microcracks in the HPC, reducing the peak value of the pore distribution at 2.5 μm. This led to a slight refinement of the pore structure but the overall porosity of the HPC samples remained relatively high, resulting in minimal improvement in the mechanical properties of the HPC samples. From day 6 to 30 of the water recuring (M-900-W-30d), the hydration of β-C_2_S formed CH and C-S-H gel, which had higher micro-mechanical properties and filled the pores. This significantly reduced the number of pores larger than 1 μm. The porosity of microcracks, large capillaries, and small capillaries decreased by 2.0%, 2.2%, and 1.2%, respectively, thereby enhancing the compressive strength of the HPC samples.

During cyclic recuring of the HPC samples damaged at 900 °C, the carbonation process could densify the HPC matrix to a greater extent than the hydration process. After 6 days of cyclic recuring, HPC samples (M-900-C-6d) exhibited a significant reduction in the pore distribution peak at 0.12 μm (Figure 11c), with the porosity of microcracks and large capillaries decreasing by 4.7% and 18.0%, respectively. This led to a high degree of compactness in the HPC matrix. The formation of AFm and AFt in the HPC samples reduced pore diameters, and the carbonation process produced calcium carbonate, which further filled the interstitial pores among AFm and AFt crystals as well as the remaining coarsened pores and microcracks, significantly increasing the compactness of the HPC samples. After 30 days of cyclic recuring, a minor pore distribution peak was present only at 17 nm in the HPC samples (M-900-C-30d-O), and the total porosity decreased by 5%. During cyclic recuring of 900 °C thermally damaged HPC samples, the ongoing processes of rehydration and carbonation contributed to the increase in matrix compactness, further enhancing the compressive strength of the HPC samples.

### 3.5. Discussion

The compressive strength of the HPC samples showed notable recovery under both recuring regimes, with cyclic recuring showing significantly greater strength recovery compared to water curing [22].

After exposure to 600 °C, a small portion of the C-S-H gel in the HPC underwent complete decomposition [14,40], while C_3_S remained largely preserved due to its limited solid-phase reaction with SF at this temperature [46,47]. During water recuring, Ca^2+^ and OH^−^ ions from saturated lime water diffused into the HPC through microcracks. Concurrently, ions leached from cement particles, particularly C_3_S, causing supersaturation in the pore solution and promoting CH formation within the microcracks and coarsened pores. The C-S-H gel, which lost only physically bound water, rapidly rehydrates during recuring, leading to volumetric expansion [38]. Rehydration and absorption effectively filled the microcracks and coarsened pores, rapidly enhancing the mechanical properties of the HPC samples. By the end of a 3-day water curing period, HPC achieves compressive strength comparable to that of undamaged HPC. During cyclic curing, CO_2_ infiltrated the microcracks, reacting with Ca^2+^ ions in the pore solution to precipitate CaCO_3_. This process reduced the ion levels, facilitating leaching of Ca^2+^ ions from cement particles and CH [41]. The C-S-H gel also underwent decalcification [42,59], evidenced by decreased amorphous phases from day 0 to 6. The crystallization and precipitation of CaCO_3_ within the microcracks and paste significantly enhanced the mechanical properties of the HPC samples, resulting in a 10.1% increase in the compressive strength for M-600-C-6d compared to M-600-W-6d (Figure 3b). From day 6 to 18, the rate of product formation in HPC decreased. The SEM and MIP results confirmed that the initial recuring period was critical for microstructure repair. After 18 days, the compressive strength recovery of the HPC samples under both water and cyclic recuring surpasses 95% of the total recovery seen after 30 days; therefore, 18 days can be considered the optimal recuring duration for HPC damaged at 600 °C.

After exposure to 900 °C, most of the C-S-H gel in the HPC completely decomposed, leaving a significant amount of β-C_2_S distributed throughout the paste [14,31]. Due to severe damage to the compactness of the HPC matrix and the development of numerous coarse pores within the paste, ions in the HPC pore solution reacted extensively with SF originally embedded in the C-S-H gel during water recuring. This led to the reduced crystallization of CH in the early recuring stages and higher production of the C-S-H gel. The C-S-H gel primarily precipitated within the cement paste, while AFm and AFt crystals predominantly formed within the microcracks [22,60]. Due to the high porosity of the HPC samples exposed to 900 °C, the limited amount of AFm and AFt cannot effectively fill the microcracks and coarse pores [61], resulting in minimal improvement in the compressive strength during the initial 0 to 6 days of water recuring. As continuous hydration of β-C_2_S progresses, CH and additional C-S-H gel are generated, effectively filling microcracks and coarse pores and significantly enhancing the compressive strength of HPC samples during this stage. During cyclic curing, CO_2_ permeated the pore solution and reacted primarily with Ca^2+^ ions leached from C_2_S to form CaCO_3_ and silica gel. This accelerated the reaction rate of β-C_2_S in the HPC, with CaCO_3_ precipitating and filling voids between AFm and AFt in microcracks and cement paste, refining the pore structure during the initial carbonation phase (3 to 6 days). Compressive strength notably increased during this stage; however, from the 6th to 18th day of cyclic recuring, the rate of rehydration and carbonation product generation in HPC significantly decreased, resulting in a slower rate of pore filling and compressive strength growth. The compressive strength of the HPC samples exhibited a slow growth trend during the 30-day water recuring period, whereas cyclic recuring not only enhanced the level of compressive strength recovery but also accelerated its rate of strength recovery. The optimal cyclic curing period for 900 °C thermally damaged HPC is 18 days.

**Figure 12:**
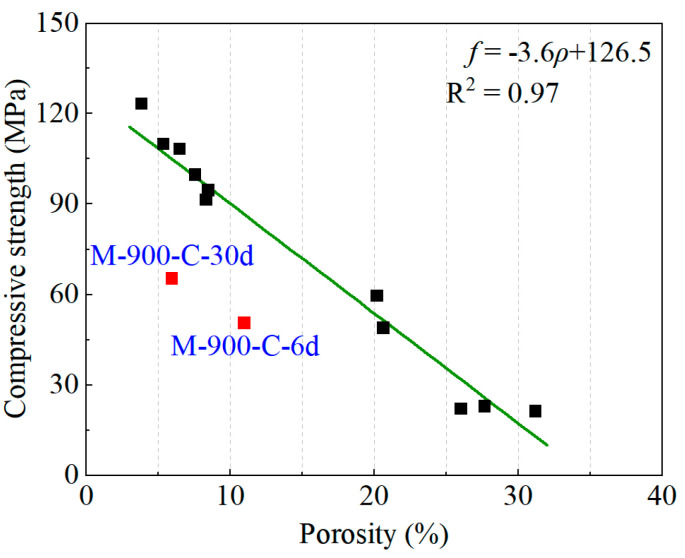
The relationship between porosity and compressive strength of HPC samples.

Based on the mechanical property and microscopic test results detailed in Section 3.1 to Section 3.4, the impact of recuring products in the pore structure of the HPC samples and the recovery of compressive strength exhibited a strong correlation. A quantitative correlation fitting of compressive strength and porosity of the HPC samples was conducted, depicted in Figure 12. Consistent with prior research [14,26,31], there is a robust linear relationship between porosity and the compressive strength of HPC samples. The deviation of 900 °C thermally damaged HPC under cyclic recuring from the trend line in Figure 12 may stem from variations in carbonation depth. This suggests potential discrepancies in porosity between the surface and the core of the HPC samples. Consequently, the porosity and compressive strength data for M-900-C-6/30d were excluded from the trend line fitting.

## 4. Conclusions

This study explored the compressive strength recovery process of high-performance concrete (HPC) subjected to thermal damage and subsequently treated using water and water–CO_2_ cyclic recuring methods. A range of characterization techniques, including X-ray diffraction (XRD), scanning electron microscopy (SEM), and mercury intrusion porosimetry (MIP) were employed to analyze the phase composition and microstructure of HPC samples at various stages: before heating, after heating, and during the recuring period. This study identified optimal recuring regimes and durations for HPC subjected to different levels of thermal damage, elucidating the underlying mechanisms of HPC recuring recovery.

1. Both water and water–CO_2_ cyclic recuring methods can effectively improve the compressive strength of thermally damaged HPC. Cyclic recuring can accelerate the recovery rate of compressive strength of HPC and improve the overall degree of strength recovery. The optimal recuring period for HPC samples exposed to 600 and 900 °C under cyclic recuring conditions is 18 days;
2. The primary reason for the compressive strength recovery of thermally damaged HPC is the filling of microcracks and coarsened pores, which increases matrix density. The analysis of HPC porosity and compressive strength reveals a strong linear correlation between the two variables;
3. Compared to water recuring, the formation of carbonation products such as CaCO_3_ and silica gel within the microcracks and cement paste in HPC under cyclic recuring can indeed further enhance its compressive strength. The amounts of rehydration and carbonation products follow the same trend as the development of compressive strength and matrix density.

For future research, enhancing the carbonation depth during cyclic recuring will be crucial for improving the strength recovery of thermally damaged HPC. Exploring how temperature variations unevenly affect strength recovery across cross-sections during actual component damage and recovery processes will enhance the application of recuring methods in practical engineering contexts.

## Figures and Tables

**Table 1:** The mix proportions of the HPC samples (unit: kg/m^3^, the quantity of materials required to prepare 1 m^3^ of mortar).

Mix Design	Cement	Silica Fume	Silica Flour	Fine Aggregates	Superplasticizer	Water	Polypropylene Fibers
Mortar	872.4	87.2	270.5	820.1	6.1	345.5	3.0

**Table 2:** The chemical compositions of the cement and silica fume.

	CaO	SiO_2_	Al_2_O_3_	Fe_2_O_3_	MgO	K_2_O	TiO_2_	SO_3_	SrO
Cement (%)	61.78	20.56	5.13	3.57	3.76	0.59	0.23	3.98	0.03
Silica fume (%)	0.11	97.70	0.16	0.07	0.44	0.23	-	0.99	-

**Table 3:** The particle size distribution of the materials (unit: μm).

Materials	D10	D50	D90
Cement	1.26	11.91	37.81
Silica fume	0.48	0.62	0.80
Silica flour	58.05	112.51	170.71
Sieved standard quartz sand	148.85	343.72	842.42

**Table 4:** The technical properties of polycarboxylate superplasticizer.

Form	Color	Density	Effective Content	pH	Total Cl^−^	Alkali Content	Water Reducing Rate
Liquid	Yellow	1.1 g/mL	24.7%	5.4	≤0.2%	≤4.0%	≥20.0%

## Data Availability

Data are contained within the article.
